# EFFECT OF TLR4 INHIBITION IN FAT-INDUCED INSULIN RESISTANCE IN HUMAN SUBJECTS

**DOI:** 10.1093/geroni/igz038.393

**Published:** 2019-11-08

**Authors:** Hanyu Liang, Nattapol Sathavarodom, Claudia Colmenares, Vinutha Ganapathy, Beverly Orsak, Nicolas Musi

**Affiliations:** 1 Barshop Institute for Longevity and Aging Studies, UT Health San Antonio Geriatric Research Education Clinical Center, STVHCS, San Antonio, Texas, United States; 2 UT Health San Antonio, San Antonio, Texas, United States; 3 Barshop Institute for Longevity and Aging Studies, University of Texas Health Science Center, San Antonio, Texas, United States

## Abstract

Older and obese subjects have increased levels of free fatty acids (FFA) in plasma that may mediate the inflammation and insulin resistance seen in these individuals. Data generated mostly in cells and rodents suggest that toll like receptor 4 (TLR4) mediates the inflammatory and insulin resistant states induced by FFA. In the present study, we tested the hypothesis that pharmacologic blockade of TLR4 would prevent lipid-induced insulin resistance. We recruited 10 lean, healthy subjects (Age: 51 ± 1 y, Sex: 6M/4F, BMI: 23.8 ± 0.7 kg/m2, Fasting plasma glucose (FPG): 5.5 ± 0.1 mmol/l). They were randomized to receive the following 72 h long i.v. treatments on separate occasions: saline (30 ml/h)+placebo (12 mg every 12 h); Intralipid (30 ml/h)+placebo; Intralipid (30 ml/h)+eritoran (12 mg every 12 h). After these infusions, insulin sensitivity was measured with an hyperinsulinemic clamp. Infusion of Intralipid significantly decreased insulin sensitivity (M value) by 14%. FPG and fasting plasma insulin concentrations increased with Intralipid infusion by 7% and 22%, respectively. Intralipid also caused a low-grade inflammatory state, evidenced by increases in plasma levels of TNFa (32%), lipopolysaccharide (14%), LPS binding protein (21%), and blood monocytes counts (15%). However, metabolic and inflammatory outcomes were not different between the Intralipid+placebo and the Intralipid+eritoran groups. We conclude that short-term TLR4 inhibition with eritoran fails to prevent lipid-induced inflammation and insulin resistance. Studies with longer acting TLR4 inhibitors may be needed to clarify the role of TLR4 on the pro-inflammatory and insulin resistant states seen with aging and obesity.

